# Extended Reality Applications in Otolaryngology Beyond the Operating Room: A Scoping Review

**DOI:** 10.3390/jcm13216295

**Published:** 2024-10-22

**Authors:** Stefan R. Torborg, Maxwell P. Kligerman, Marc Cohen, Javin Schefflein

**Affiliations:** 1 Cancer Biology and Genetics Program, Sloan Kettering Institute, Memorial Sloan Kettering Cancer Center, New York, NY 10065, USA; 2 Weill Cornell/Rockefeller/Sloan Kettering Tri-Institutional MD-PhD Program, New York, NY 10065, USA; 3 Department of Surgery, Memorial Sloan Kettering Cancer Center, New York, NY 10065, USA; 4 Department of Radiology, Memorial Sloan Kettering Cancer Center, New York, NY 10065, USA

**Keywords:** extended reality, multidisciplinary planning, otolaryngology, patient education, preoperative planning

## Abstract

**Objective**: Extended reality (XR) has increasing usage in medicine, especially surgical fields, but the scope of applications is largely limited to intraoperative navigation. The aim of this scoping review is to evaluate all the available literature on how XR technologies have been applied to otolaryngology—head and neck surgery (OHNS) beyond the operating room for applications such as patient education and interdisciplinary communication. **Review Methods**: Using the Preferred Reporting Items for Systematic reviews and Meta-Analyses extension for Scoping Reviews (PRISMA-ScR) guidelines, we systematically searched PubMed and Scopus. Studies were reviewed without temporal restriction. Inclusion criteria comprised English-language, peer-review papers or conference abstracts studying XR technologies for non-operative uses such as patient education, physician training, or interdisciplinary preoperative planning in the field of OHNS. **Results**: Database searching initially identified 1607 records. After filtering for duplicates, screening for relevance, and applying the inclusion criteria, 10 studies were ultimately included. This study identifies gaps in the existing literature and describes future applications and key areas of research. XR is a novel strategy for increasing patients’ comprehension of their procedures and can facilitate improved communication and planning amongst a multidisciplinary surgical team during preoperative discussions. However, the existing literature is small in scale and has low statistical power for demonstrating clinical benefits. **Conclusions**: More robust studies are required to determine the true value of implementing XR. XR is a promising new technology with potential to advance education and patient care in OHNS. Ongoing research will continue to optimize the use of XR technology, ensuring safe and effective integration into clinical practice.

## 1. Introduction

Extended reality (XR) is an emerging technology that encompasses virtual reality (VR), augmented reality (AR), and mixed reality (MR) to create immersive and interactive experiences [1]. These technologies integrate the physical stimuli of vision, hearing, and touch with a simulated environment that can be fully immersive (VR), overlayed on the real world (AR), or a combination of both (MR) [1]. In the field of medicine, XR has great potential to transform the way healthcare professionals learn, train, and provide patient care. It has gained attention in recent years as a promising tool in medical education, patient communication, and surgical planning. For example, VR platforms can provide an immersive environment for students to study anatomy [2], or a controlled environment for surgical trainees to learn and practice complex procedures [3]. AR and MR have numerous applications in surgery, such as providing surgeons with patient-specific anatomical data during cases to improve the efficacy, speed, and safety of operations [4,5]. However, many of these applications remain understudied.

One field where XR can improve patient care is otolaryngology—head and neck surgery (OHNS). In OHNS, XR can improve how physicians approach complex surgical procedures and interact with patients by simulating the complex anatomical features of the head and neck system and providing a better understanding of the various structures and their interconnections [6]. Medical professionals of all levels can use XR to enhance their training in OHNS, allowing them to practice complex procedures in a virtual environment before performing them on patients [7]. In recent years, there has been an explosion of interest in applying XR for these purposes. In addition, XR can be used to educate patients about their conditions and treatment options, as it provides an immersive and interactive platform to demonstrate complex medical procedures and conditions to patients [8]. This environment can provide them with a better understanding of their options, improving patient medical literacy and decision-making. Such patient-focused applications have yet to be thoroughly explored. As XR technology continues to advance, it is likely that its use in OHNS and other medical specialties will become increasingly widespread. This scoping review aims to summarize the existing literature on XR in OHNS, highlight understudied areas of XR with potentially significant impacts on medicine, and provide an introduction for clinicians interested in further exploring the potential uses of this technology in medicine.

## 2. Methods

**Figure 1:**
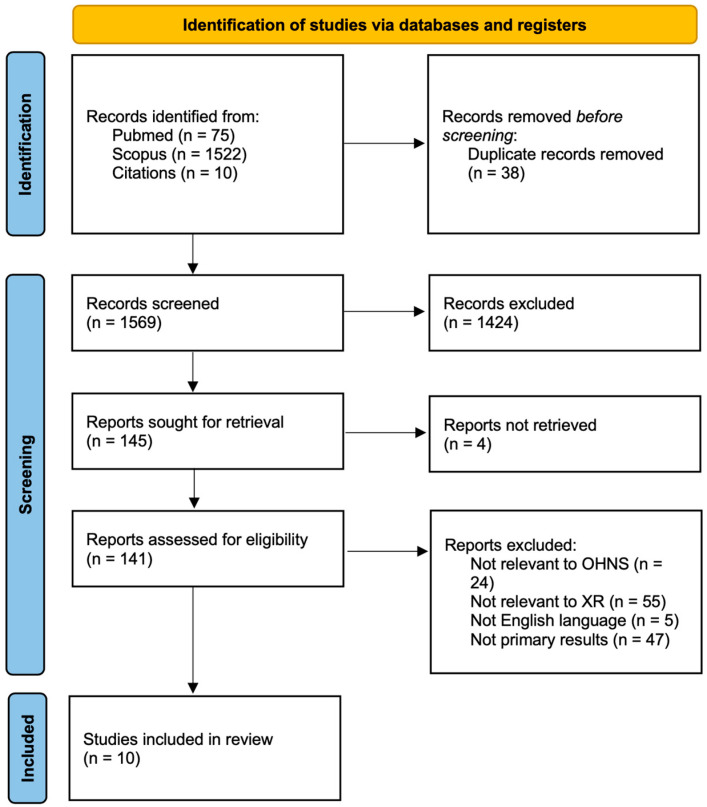
Database search strategy: PRISMA flow diagram of study identification, screening, and article inclusion.

A scoping review was conducted using the Preferred Reporting Items for Systematic reviews and Meta-Analysis extension for Scoping Reviews (PRISMA-ScR) (Figure 1). The databases searched were PubMed and Scopus. The specific search terms used were phrases of the research topics (patient education, preoperative planning, and simulation training), the technology (extended reality, augmented reality, and virtual reality), and the field (otolaryngology, head and neck surgery, and skull base), separately and in combinations using Boolean operators. All searches were carried out on 8 June 2024, and no restrictions on publication date were applied. Inclusion criteria consisted of English-language, peer-review papers and conference abstracts utilizing XR technologies in OHNS for applications outside of the operating room. This broadly covered topics such as patient education, simulation, operative planning, and training while excluding studies on intraoperative visualization and navigation. Additional studies were manually screened from the references of included studies and articles not containing primary research such as reviews.

Data were extracted from the included articles using a predefined data summary sheet to standardize the information collected (Supplementary Table S1). The data extracted included general paper characteristics (first author, year of publication, and journal), study subject (e.g., physicians or patients), number of subjects included, type of technology (VR, AR, or MR), purpose of the study (education, surgical planning, or feasibility study), study design (number of cases included), outcomes measured, and data type (qualitative vs. quantitative results). The goals of each study were also summarized and sorted into 4 broad categories: education (student education and physician training), planning (pre-operative discussion, surgical rehearsal, and intra-operative assistance), feasibility (retrospective testing of XR and acceptance by physicians), and patient-focused (quality of care and subjective experience). If articles discussed multiple categories, all were included. The table also summarizes the key findings of each study included in this review.

## 3. Results

This review broadly summarizes the existing literature on XR utilization in OHNS, with the goal of identifying the uses of different XR modalities for patient education, physician training, or interdisciplinary preoperative planning (Supplementary Table S2). While the rate of publications studying XR in OHNS has rapidly increased in recent years, most articles study the technology for intraoperative visualization and assistance [9]. The general purpose of this application is to guide surgeons during procedures by highlighting key anatomical structures, rendered from 2-dimensional imaging such as MRI or CT into 3-dimensional holographs overlayed on the patient’s anatomy [10]. Such uses are important advances to surgical practice and have recently been thoroughly reviewed elsewhere [9,10]. Other reviews have discussed this technology in OHNS, providing in-depth analysis on studies using specific modalities such as VR or individual operative procedures [11,12]. Here, this review aims to discuss a wider scope of XR technologies and applications for OHNS.

### 3.1. Preoperative Applications

One method of XR preoperative planning is the creation of anatomically accurate 3-dimensional models from preoperative patient imaging data. For example, Filimonov et al. developed five patient-specific models in VR from CT and MRI imaging to assist in planning an endoscopic endonasal approach to craniovertebral junction surgery [13]. Surgeons manipulated these models in VR, including tissue removal and the insertion of various surgical instruments, to simulate different dissection trajectories. Similarly, Mitani et al. developed a case-specific MR simulation from CT data to assist in preoperative planning [14]. The system was synced across headsets so that multiple surgeons could simultaneously view the MR model, both preoperatively and intraoperatively. The surgeons used the model to discuss the surgical approach. Such models provide a realistic visual environment for examination of patient-specific anatomy that can simultaneously be used by multiple physicians. This visualization can preoperatively identify anatomical variations that require changes in surgical approach, leading to decreased surgical times and complications.

XR models derived from patient imaging also benefit surgical training. The anatomical differences across patients provide a variability that is important for medical trainees to observe and practice on. Arora et al. used CT scans of 24 cadaveric temporal bones to develop generic and patient-specific VR models [15]. Participants were tasked with performing an extended cortical mastoidectomy, posterior tympanotomy, and cochleostomy on both the VR and physical temporal bones. Yamazaki et al. created VR and MR models of healthy temporal bone for anatomical training and studied one case of a petrous apex cholesteatoma for preoperative planning and intraoperative assistance [16]. By incorporating normal anatomical variability into models of surgical education, medical trainees can observe and practice on a wider range of anatomy at higher throughput and in lower-stakes environments. Practicing procedures in anatomically accurate and varied simulations also reduces the need for cadaveric training material and increases the amount of training that individuals can obtain.

Additional studies produced patient-specific XR models for training and planning but focused on the quality and development of the systems involved. Rose et al. developed a pipeline for the integration of CT and MRI into an AR platform, which was then used in a face validity study to test the anatomical accuracy and overall utility of the system [17]. Won et al. used a VR platform to render the anatomy of 10 patients who had already undergone various skull base surgeries from CT scans [18]. Surgeons evaluated the models by watching videos of the actual procedure and recreating key steps of the surgery in VR. Such studies are essential to ensure the accuracy and realism of simulated environments, without which the benefit to training and planning is lost.

### 3.2. Multidisciplinary Planning

**Figure 2:**
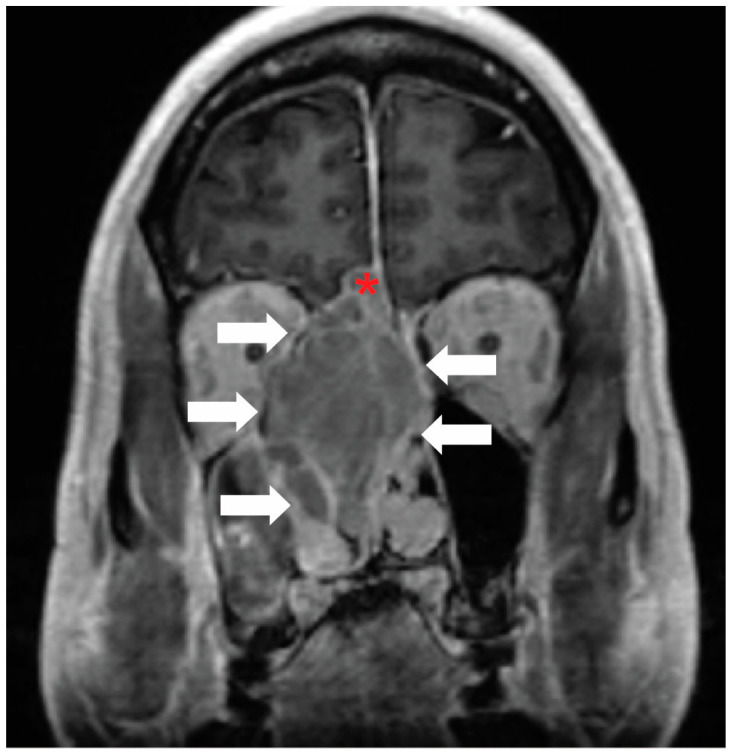
Classical tumor visualization: Contrast-enhanced coronal MRI of the face demonstrates an esthesioneuroblastoma in the right ethmoid air cells (white arrows). There is also intracranial extension (red asterisk). Image and annotations are from the authors.

**Figure 3:**
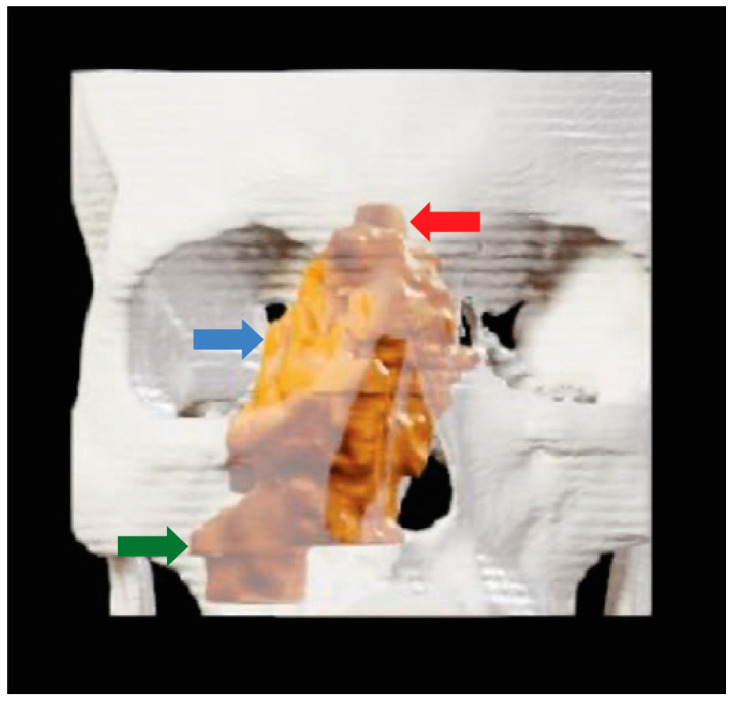
Tumor visualization with XR: 3-dimensional reconstruction of the esthesioneuroblastoma (orange) over 3-dimensional skull segmentation performed by the authors. The tumor extends into the right maxillary sinus (green), right medial orbit (blue), and right anterior cranial fossa (red). The reconstruction demonstrates how 2-dimensional scans can be processed into 3-dimensional objects to visualize the anatomical proximity of various structures preoperatively.

**Figure 4:**
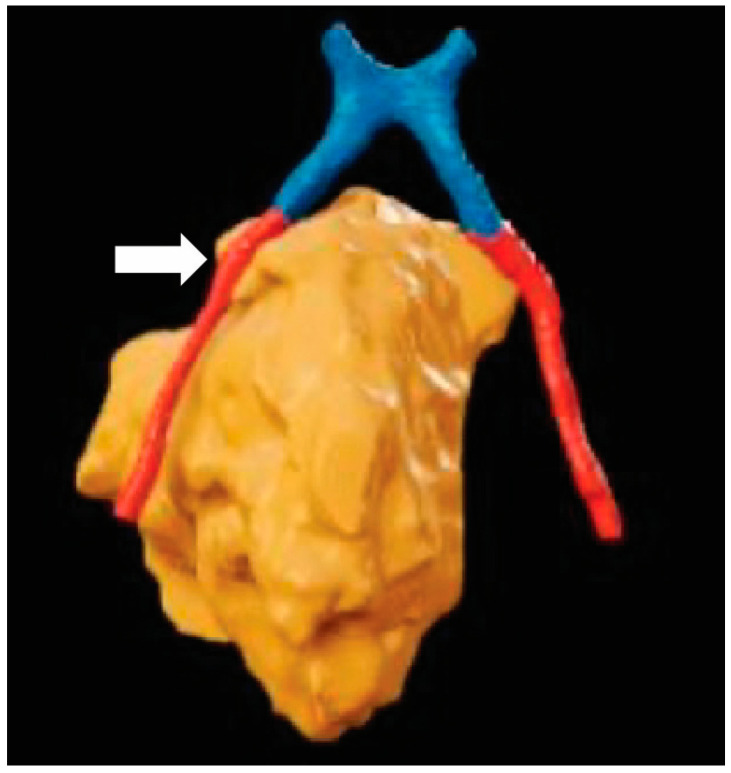
Surgical planning with XR: A top-down 3-dimensional reconstruction of the optic chiasm (blue), optic nerves (red), and esthesioneuroblastoma (orange). Although the right optic nerve approximates the tumor, there is no direct invasion (white). The reconstructed objects allow for clear visualization of the spatial relationship between anatomical structures that may impact surgical plans.

Although there are many possible applications for surgeons to use XR, focusing solely on surgeons greatly limits the scope of applications for XR. Specifically, there is great potential in including radiologists, medical oncologists, radiation oncologists, and other specialties in preoperative planning using XR. Many OHNS cases are already discussed in tumor board formats where multiple specialties are present. Adapting XR technology to these sessions can greatly enhance surgical decision-making. In the existing OHNS literature, all six published papers that studied XR technologies using physicians exclusively studied surgeons, with no inclusion of other medical specialties. The remaining papers focused on medical students in an educational environment. At our institution, standard MRI imaging (Figure 2) was used by the Neuroradiology faculty to generate 3-dimensional segmentation for visualization (Figure 3) in conjunction with the Otolaryngology department to better evaluate an esthesioneuroblastoma for surgical optimization (Figure 4). Using XR technology can facilitate communication and planning across medical disciplines and optimize the decision-making process.

### 3.3. Patient Interaction

One study evaluates the role of XR for directly interacting with patients. Caruso et al. studied AR for reducing anxiety and improving the cooperation of pediatric patients during minor outpatient OHNS procedures [19]. Patient satisfaction ratings of AR were high, but the study only included three patients and self-reported ratings of fear (Children’s Fear Scale [20]) and pain (Numeric Rating Scale [21]) were initially low and remained largely unchanged post-procedure. The limited existing literature highlights many fundamental gaps in our understanding of XR applications in OHNS. XR has potential to improve medical care when interacting with patients, such as increasing medical literacy and knowledge by explaining complex surgical procedures in an immersive, visual environment.

## 4. Discussion

These published studies represent important early steps in developing and understanding the uses of XR in OHNS. However, the existing literature remains limited in both scope and scale. Many studies are proof-of-concept, aiming to demonstrate the feasibility of XR and the possible applications of the technology. This review finds little research to date using actual patient data. Most papers have created XR algorithms from simulated or cadaveric models. For example, Rose et al. created a highly accurate AR simulation of thyroid and neck anatomy, but the rendering was developed from a plastic model and patient data were not used in the study [17]. Applying these technologies to patient data, even if not directly for patient care, represents an important next step. As Arora et al. used cadaveric temporal bones, there was no opportunity to apply VR technology to preoperative planning for living patients [15]. Furthermore, in qualitative analysis, most anatomical structures were not rated by OHNS specialists as adequate (Likert score < 4 [22]). However, the inclusion of matched VR and cadaveric models in the surgical tasks did allow for patient-specific comparisons of surgical performance and VR model quality. When compared to the generic VR model, VR models from individual patients scored worse in anatomical accuracy, likely due to lower resolution, but scored higher in utility for surgical planning. This highlights the benefits of individualized VR renderings, providing detail on patient-specific anatomical variations.

Another limitation is the use of retrospective patient data. Assessment of XR is not only important for accuracy, but also for compatibility with existing hospital workflows. With retrospective data, the workflow from image acquisition to implementation is not assessed. In Filimonov et al., five patient cases were used to render VR environments, but the surgeries were already completed, and the simulations were only used to test the method feasibility [13]. Additionally, no analysis was performed to quantify the impact of VR on surgical outcomes. Similarly, Won et al. accurately reproduced patient-specific anatomy from 10 patients across a wide range of skull base surgeries, highlighting the potential of VR to simulate varied anatomical and pathological features [18]. However, all cases were completed surgeries and were selected to demonstrate feasibility. Use of retrospective studies may indicate a selection bias, as the chosen cases may not represent an even spectrum of patients or diseases. To be practically useful, XR tools must integrate into current hospital workflows and be compatible with a wide range of anatomy and pathophysiology.

Most publications also included few clinical examples, often with only a single patient. Although these studies were thorough, they are not generalizable. In Yamazaki et al., XR was integrated longitudinally in an individual case [16]. First, VR was used to study patient-specific anatomy and plan a surgical approach. Second, MR was integrated into the OR, assisting in identifying key anatomical structures. This study demonstrates the thorough and successful integration of XR into patient care, but was only performed on one patient. Although Mitani et al. successfully demonstrated the use of MR in preoperative planning involving the collaborative discussion of multiple physicians, this was only carried out with one patient [14]. Such publications are an important first step, exploring potential uses for XR and establishing basic principles for implementing the technology. However, in-depth research studying a wider patient population is required to ensure the generalizability of XR and establish the benefits of XR for patient care.

Another gap in the existing literature is that there is little quantitative analysis on the benefits of XR technology. For example, Arora et al., Rose et al., Yamazaki et al., and Mitani et al. use qualitative metrics to demonstrate that XR technologies are seen by physicians as beneficial to patient care by improving confidence, enjoyment, and engagement, and for training [14,15,16,17]. The remaining studies do not include any formal analysis. While promising, these results do not demonstrate any tangible benefits to patient care. Another concern regarding these metrics is that the physicians willing to participate in such studies may be biased towards individuals with a greater interest in using XR in medicine or ability to incorporate the technology. This bias may overestimate the utility of XR and the ease of its use and integration into existing medical procedures.

Although physician ratings on the ability of XR to improve patient care are overwhelmingly positive, there is significantly less data on XR improving quantitative metrics such as surgical time and patient outcomes. The few studies with quantitative results for XR are performed only in educational settings and do not address patient care. Additionally, only one study demonstrates a quantitative improvement with XR. Specifically, Malik et al. studied MR for improving medical student education in neck anatomy [23]. Students using MR asked more questions than students in traditional lectures, but overall engagement was not improved. Baseline quiz performance was equal between groups, but students using MR performed significantly better in the post-intervention quiz than students in the traditional teaching session. This result is promising for XR in educational settings, but the benefit to patient care remains unknown.

Other quantitative studies, in addition to being in an educational setting, suffer from too small a sample size to demonstrate statistically significant benefits of XR. For example, Chen et al. compared VR simulation to traditional cadaveric studies or 2-dimensional anatomy atlases for medical students learning skull anatomy [24]. There was no difference between the groups in pre-intervention tests. Although the VR and cadaveric groups had higher subjective ratings of enjoyment, authenticity, and learning efficiency compared to the atlas group, there was no difference in the post-intervention tests either. Furthermore, the VR group did not have higher subjective ratings than the traditional cadaveric group. Similarly, Talks et al. studied the ability of VR to reduce procedure time [25]. Medical students completed VR simulated cortical mastoidectomies before being separated into two groups to perform the procedure on 3-dimensional printed models, either on models of cases they had studied in VR or on new models. There was no difference in procedure duration and surgical performance (Melbourne Mastoidectomy Scale [25]) between the two groups, although there was a wide range in time and score. One hindering factor was that all participants were medical students with no operating experience, likely contributing to the large variance in the data. Both studies were small in scale and produced ambivalent results, suggesting that the benefits of XR to education may be modest and require more concrete, quantitative analysis to identify. Future XR studies must closely consider the optimal format for testing and outcome quantification.

The results of this review demonstrate the promising future of using XR technology to improve multiple aspects of patient care in OHNS. XR has many novel applications for different groups, which despite not being fully developed are nonetheless exciting. Training for surgical residents and faculty on realistic and variable simulation models will increase familiarity with the natural anatomic differences between patients, improving trainee skill and confidence. Creating easily understandable and accessible visual models will facilitate patients learning about their personal health, contributing to an improvement in health literacy that is desperately needed. The easily approachable, visual nature of XR will allow for improved communication between individuals with different knowledge and expertise, including both patient–doctor and interdisciplinary communication. However, outside the scope of this review, XR also has many uses intraoperatively, such as the concurrent overlay of 3-dimensional reconstructions over the patient’s anatomy, allowing surgeons to “see through” the patient and make real-time adjustments to their surgical approach. As displayed in this review, the potential for XR to integrate with and improve nearly every aspect of medical care demonstrates its high promise.

This review highlights many exciting and understudied features of XR applications in OHNS. However, there are some limitations to this study. The number of publications on XR applied to education, training, and planning are very limited and on diverse subjects. Due to this topic diversity, the specific search strategy used may have left out articles despite the extensive set of combinations used. Additionally, the small number of relevant publications makes it difficult to draw strong conclusions about the state of the field. These concerns will be addressed as the literature on XR in OHNS expands.

## 5. Conclusions

XR technology is a promising new avenue for advancing multiple aspects of medical care in OHNS. By providing immersive and interactive experiences, XR can help patients better understand their conditions and treatment options, leading to improved patient satisfaction, outcomes, and medical literacy. This review highlights how the integration of XR technology into clinical practice has the potential to increase patient education in OHNS and improve overall healthcare outcomes. Furthermore, the analysis performed here shows that most applications of XR in surgical planning are limited in scope and solely involve surgeons. Critical medical decisions, such as whether to pursue surgery, are made in collaborations between patients, surgeons, radiologists, medical oncologists, and other specialties. The studies discussed in this review suggest that integrating XR into this discussion process can change medical decision-making, improving both quality of care and patient experience. However, most studies on XR are preliminary in nature, focused solely on proof-of-concept technology development or lacking sufficient size to make strong and generalizable conclusions. These studies are critical early steps in the development of XR in OHNS, but this review establishes that significant further steps are necessary before the technology can be reliably and safely integrated into medical practice.

For clinicians and healthcare organizations interested in integrating XR technology into medical practice, it is important to also consider practical aspects of XR applications. In all situations, there is an upfront cost to purchase XR equipment and the development of virtual models requires a moderate time investment by an individual trained in XR software. Individuals should explore various software and hardware options to determine which is most suitable to the desired application. Whether XR will be used for medical training or direct patient care is another important consideration. Use in patient care implies a much greater scale of throughput, demands fast turnaround times, and requires coordination between radiologists, XR technicians, and others. Protocols for developing simulations from patient imaging data must also be standardized to ensure accuracy and reproducibility. Moving forward, further research and development in applying XR technology will be critical to unlocking its full potential for enhancing medical care.

## Data Availability

Data that support the findings of this study are available on request from the corresponding author. The data are not publicly available due to privacy or ethical restrictions.

## Supplementary Materials

The following supporting information can be downloaded at: https://www.mdpi.com/article/10.3390/jcm13216295/s1, Supplementary Table S1: Study characteristics, Supplementary Table S2: Uses of extended reality in otolaryngology-head and neck surgery.
